# Anti-inflammatory and immunomodulatory mechanisms of mesenchymal stem cell transplantation in experimental traumatic brain injury

**DOI:** 10.1186/1742-2094-10-106

**Published:** 2013-08-23

**Authors:** Run Zhang, Yi Liu, Ke Yan, Lei Chen, Xiang-Rong Chen, Peng Li, Fan-Fan Chen, Xiao-Dan Jiang

**Affiliations:** 1 The National Key Clinic Specialty, The Neurosurgery Institute of Guangdong Province, Guangdong Provincial Key Laboratory on Brain Function Repair and Regeneration, Department of Neurosurgery, Zhujiang Hospital, Southern Medical University, Guangzhou 510282, China; 2 Department of Neurosurgery, Shenzhen Second People’s Hospital, the First Affiliated Hospital of Shenzhen University, Shenzhen 518000, China

## Abstract

**Background:**

Previous studies have shown beneficial effects of mesenchymal stem cell (MSC) transplantation in central nervous system (CNS) injuries, including traumatic brain injury (TBI). Potential repair mechanisms involve transdifferentiation to replace damaged neural cells and production of growth factors by MSCs. However, few studies have simultaneously focused on the effects of MSCs on immune cells and inflammation-associated cytokines in CNS injury, especially in an experimental TBI model. In this study, we investigated the anti-inflammatory and immunomodulatory properties of MSCs in TBI-induced neuroinflammation by systemic transplantation of MSCs into a rat TBI model.

**Methods/results:**

MSCs were transplanted intravenously into rats 2 h after TBI. Modified neurologic severity score (mNSS) tests were performed to measure behavioral outcomes. The effect of MSC treatment on neuroinflammation was analyzed by immunohistochemical analysis of astrocytes, microglia/macrophages, neutrophils and T lymphocytes and by measuring cytokine levels [interleukin (IL)-1α, IL-1β, IL-4, IL-6, IL-10, IL-17, tumor necrosis factor-α, interferon-γ, RANTES, macrophage chemotactic protein-1, macrophage inflammatory protein 2 and transforming growth factor-β1] in brain homogenates. The immunosuppression-related factors TNF-α stimulated gene/protein 6 (TSG-6) and nuclear factor-κB (NF-κB) were examined by reverse transcription-polymerase chain reaction and Western blotting. Intravenous MSC transplantation after TBI was associated with a lower density of microglia/macrophages and peripheral infiltrating leukocytes at the injury site, reduced levels of proinflammatory cytokines and increased anti-inflammatory cytokines, possibly mediated by enhanced expression of TSG-6, which may suppress activation of the NF-κB signaling pathway.

**Conclusions:**

The results of this study suggest that MSCs have the ability to modulate inflammation-associated immune cells and cytokines in TBI-induced cerebral inflammatory responses. This study thus offers a new insight into the mechanisms responsible for the immunomodulatory effect of MSC transplantation, with implications for functional neurological recovery after TBI.

## Background

Traumatic brain injury (TBI) is a major cause of mortality and morbidity among the population worldwide [1]. The inflammatory response is regarded as a key factor in the secondary injury cascade following TBI. Activation of the inflammatory cascade is mediated by the release of pro- and anti-inflammatory cytokines [2,3]. TBI induces a strong inflammatory response characterized by the recruitment of peripheral leukocytes into the cerebral parenchyma and the activation of resident immune cells [4,5]. The infiltration of neutrophils, monocytes and lymphocytes to the injured site directly affects neuronal survival and death [4-7]. Moreover, activated microglia migrate to injured sites and release cytokines, chemotactic cytokines, reactive oxygen species, nitric oxide, proteases and other factors with cytotoxic effects, which may in turn exacerbate neuronal death [6,8].

However, these immune cells and inflammatory mediators can also have neuroprotective effects in TBI [3,9]. For example, T lymphocytes may contribute to later repair processes in brain injury [10,11]; proinflammatory cytokines such as interleukin (IL)-1, IL-6 and tumor necrosis factor (TNF)-α have both deleterious and beneficial effects on neural cells [7,12,13]; and microglia can remove cell debris, promote tissue remodeling and exert numerous neuroprotective effects under certain conditions [4,14,15]. Of these, TBI-induced inflammation appears to be a key factor in secondary brain damage, which suggests that anti-inflammatory or immunoregulatory strategies could provide effective treatments for the management of TBI-induced pathology.

Previous studies have shown beneficial effects of mesenchymal stem cell (MSC) transplantation in central nervous system (CNS) injuries, including TBI, stroke and spinal cord injury animal models. The main findings of these studies suggested that MSCs improved neurological functional recovery, decreased apoptosis, increased endogenous cell proliferation, promoted angiogenesis and reduced lesion size [16]. The potential mechanisms whereby transplanted MSCs might exert beneficial effects in CNS injury include their ability to migrate to injured tissues, transdifferentiation to replace damaged neural cells and the production of growth factors by MSCs [16-18]. However, recent evidence indicates that the therapeutic effect of MSC transplantation may not be through direct cell replacement, but via modulating the host microenvironment [19]. MSCs can secrete a variety of bioactive molecules such as trophic factors and anti-apoptotic molecules, which may provide the main mechanism responsible for their therapeutic effect [20].

More recently, many studies have demonstrated that MSCs possess immunomodulatory properties [21,22]. MSCs can directly inhibit the proliferation of T lymphocytes and microglial cells, and can modulate the cytokine-secretion profile of dendritic cells and monocytes and/or macrophages [20,23-25]. MSCs are also known to inhibit basal and formyl-methionyl-leucyl-phenylalanine-stimulated production of reactive oxygen species by neutrophils [26]. In experimental autoimmune encephalomyelitis models, MSCs inhibited myelin-specific T cells and induced peripheral tolerance [27,28]. The immunosuppressive effect of transplanted MSCs has also been demonstrated in acute, severe graft-versus-host disease [29] and in multiple system atrophy [30]. In addition, MSCs can induce peripheral tolerance and migrate to injured tissues, where they can inhibit the release of proinflammatory cytokines and promote the survival of damaged cells [21]. For example, the therapeutic benefit of MSC transplantation has been observed in acute lung injury [31,32], myocardial infarction [33], acute renal failure [34], cerebral ischemia [35] and Alzheimer’s disease [36]. Furthermore, some studies have found an inflammation-modulatory function for transplanted stem cells. One study demonstrated anti-inflammatory effects of human cord blood cells in a rat model of stroke [37]. Another study reported that intravenous NSCs, administered during the hyperacute stage in stroke, could modulate innate cerebral inflammatory responses by interacting with peripheral inflammatory systems [38].

These studies indicate the feasibility of using MSCs to reduce cerebral inflammation and modulate the immune response after TBI. However, few studies have focused simultaneously on the effects of MSCs on inflammation-associated cytokines and immune cells in CNS injury, especially in an experimental TBI model. In this study, we therefore investigated the anti-inflammatory and immunomodulatory properties of MSCs in TBI-induced neuroinflammation using systemic MSC transplantation in a rat TBI model.

## Materials and methods

Sprague–Dawley (SD) rats were purchased from the Animal Experiment Center of Southern Medical University (Guangzhou, China). Animals were housed under a 12-h light/dark cycle, with food and water freely available. Animal experimental procedures were approved by the Southern Medical University Ethics Committee. All surgery was performed under sodium pentobarbital anesthesia, and all efforts were made to minimize animal suffering.

### Isolation, expansion and characterization of MSCs

MSCs were generated from the bone marrow of SD rats. Mononuclear cells were isolated by gradient centrifugation at 900 *g* for 30 min on Percoll (Invitrogen, Carlsbad, CA, USA) at a density of 1.073 g/ml. The cells were then washed twice with phosphate-buffered saline (PBS) and plated at 1 × 10^6^ cells/25 cm^2^ in culture flasks in 5 ml DMEM/F12 (1:1) with 10% fetal bovine serum. After 72 h of incubation, non-adherent cells were removed from the cultures, and fresh culture medium was added to the flasks. When the cells reached 90% confluence, adherent cells were trypsinized, harvested and expanded [39]. Expanded cells from passages three−eight were used for further testing or transplantation.

MSCs were assessed by flow cytometry analysis of CD44, CD90 and CD105, and the hematopoietic markers CD14, CD34, CD45 and HLA-DR [40]. The primary antibodies used were fluorescein isothiocyanate-conjugated anti-CD44, -CD45 and -CD105, and phycoerythrin-conjugated anti-CD14, -CD34, -CD90 and -HLA-DR. All antibodies were purchased from AbD Serotec (1:10, Kidlington, UK).

### Experimental groups

SD rats were divided into three groups: (1) sham group (21 rats); (2) TBI + saline group (52 rats); (3) TBI + MSCs group (52 rats).

### Traumatic brain injury animal models

TBI animal models were produced as previously described [41]. Adult male SD rats (220–250 g) were anesthetized with 2% pentobarbital (30 mg/kg) intraperitoneally and maintained at 37°C throughout the surgical procedure using a water-heating pad. The rats were placed in a stereotactic frame. A 6-mm-diameter craniotomy was performed over the right cortex midway between the lambda and the bregma. Injury was induced using a weight-drop hitting device (ZH-ZYQ, Electronic Technology Development Co., Xuzhou, China) with a 4.5-mm-diameter cylinder bar weighing 40 g from a height of 20 cm. Rats in the sham group were subjected to the same craniotomy procedure without cortical impact.

### MSC transplantation

Two hours after TBI, the rats were neurologically evaluated using a modified neurological severity score (mNSS) test. Rats with similar neurological severity scores (13–15 points) were randomly divided into two groups that received 4 × 10^6^ MSCs in 100 μl PBS or PBS alone, respectively, via the jugular vein.

### Behavioral testing

Behavioral testing was conducted on days 1, 3, 7, 14, 21 and 28 after TBI using mNSS tests. The mNSS test includes motor, sensory, reflex and balance tests, as described previously [42]. The mNSS test is graded on a scale of 0–18, where a total score of 18 points indicates severe neurological deficit and a score of 0 indicates normal performance; 13–18 points indicates severe injury, 7–12 indicates mean-moderate injury, and 1–6 indicates mild injury.

### Measurements of brain water content

Brain water content was measured at 72 h after TBI. Following anesthesia and decapitation, the brains were removed immediately and divided into two hemispheres along the midline, and the cerebella were removed. Ipsilateral hemispheres were placed on a pre-weighed piece of aluminum foil to give the wet weight and then dried in an electric oven at 100°C for 24 h [38]. The brain water percentage was calculated as follows: (wet weight - dry weight)/(wet weight).

### Immunohistochemistry and terminal deoxynucleotidyl transferase dUTP nick end labeling staining

At 72 h after TBI, rats were anesthetized and transcardially perfused with 100 ml cold PBS and 100 ml of 4% paraformaldehyde in 0.1 M PBS. The brains were then removed, post-fixed and paraffin-embedded, and consecutive coronal sections were cut at 5-μm intervals from bregma −2.0 mm to bregma −7.0 mm to collect the entire lesioned cortex. For immunohistochemistry, slides with brain sections were deparaffinized and boiled in 10 mM citrate buffer (pH 6.0) in a microwave to expose the antigens and then blocked with 10% normal goat serum. Slides were incubated with primary antibody against glial fibrillary acidic protein (GFAP) (rabbit polyclonal to GFAP, 1:200), microglia/macrophage-specific calcium-binding protein (goat polyclonal to Iba1, 1:100, Abcam, New Territories, HK), myeloperoxidase (MPO) (rabbit polyclonal to MPO, 1:100, Abcam) and CD3 (rabbit polyclonal to CD3, 1:100, Abcam) at 4°C overnight. Following primary antibody incubation, slides were incubated in biotin-conjugated anti-rabbit IgG or anti-goat IgG (1:100, Boster, Wuhan, China), then treated with an avidin-biotin-peroxidase system (Boster) (negative controls for immunostaining was secondary antibody only). Finally, slides were stained with diaminobenzidine, and the nucleus was counterstained with hematoxylin. Terminal deoxynucleotidyl transferase-mediated dUTP nick 3′-end labeling was performed to detect dying cells using an In Situ Cell Detection Kit (Roche, South San Francisco, CA, USA) according to the manufacturer’s instructions. The number of positive cells near the injured areas was counted (8 to10 sections per brain, 500 μm apart) in a blinded manner.

### Cytokine analysis

To measure cytokine levels, rats were killed at 12, 24 and 72 h after TBI or sham operation. Brains were immediately collected, and punch biopsies (5 mm diameter) of the injured cortex were isolated and stored at −80°C. The tissue was homogenized in chilled extraction buffer containing Tris–HCl (50 mmol/l, pH 7.2), NaCl (150 mmol/l), 1% Triton X-100 and 1 mg/ml protease inhibitor cocktail (Biovision, Mountain View, CA, USA) at a ratio of 1:10 (tissue: buffer), shaken for 90 min on ice, centrifuged at 12,000 *g* for 15 min at 4°C and frozen at −80°C [43]. Total protein concentrations were measured using a BCA Protein Assay Kit (Thermo, Rockford, IL, USA). The levels of 12 cytokines [IL-1α, IL-1β, IL-4, IL-6, IL-10, IL-17, tumor necrosis factor (TNF)-α, interferon (IFN)-γ, RANTES, macrophage chemotactic protein (MCP)-1, macrophage inflammatory protein (MIP)-2 and transforming growth factor (TGF)-β1] were determined in 1 mg of total protein using the Rat Bio-Plex Pro Assays (Bio- Rad, Hercules, CA, USA), according to the manufacturer’s instructions.

### Quantitative real-time polymerase chain reaction

Total RNA was extracted from injured brain tissues at 12, 24 and 72 h after TBI using TRIzol Reagent (Invitrogen), according to the manufacturer’s instructions. Levels of TNF-α stimulated gene/protein 6 (TSG-6) and nuclear factor (NF)-κB mRNA were quantitated using an ABI 7500HT Fast Real-Time PCR System (Applied Biosystems, Grand Island, NY, USA). Glyceraldehyde-3-phosphate dehydrogenase (GAPDH) was used as an endogenous control. Sequence-specific primers for the above genes were designed using Premier 5 software as follows:

TSG-6-up: GCAGCTAGAAGCAGCCAGAAAG,

TSG-6-dn: TTGTAGCAATAGGCGTCCCACC;

NF-κB-up: CTACACTTAGCCATCATCCACCTT,

NF-κB-dn: AGTCCTCCACCACATCTTCCTG;

GAPDH-up: AAGGTGAAGGTCGGAGTCAA,

GAPDH-dn: AATGAAGGGGTCATTGATGG.

The 2^-ΔΔCt^ method was used to calculate the relative expression levels.

### Western blot analysis

Total protein was isolated from rat injured brain tissues using ice-cold RIPA buffer. Total protein concentrations were measured with the BCA Protein Assay Kit (Thermo). Protein samples (30 μg per lane) were separated using sodium dodecyl sulfate-polyacrylamide gel electrophoresis and transferred to polyvinylidene difluoride membranes. Proteins were detected by incubation with primary antibodies (mouse polyclonal to TSG-6 and mouse anti-NF-κB p65 1:250, BD Biosciences, San Jose, CA, USA) followed by secondary antibodies (goat anti- mouse IgG, horseradish-peroxidase conjugate, 1:1,000, Sigma Aldrich, St. Louis, MO, USA). Immunoblots were visualized using a Millipore ECL Western Blotting Detection System (Millipore, Billerica, MA, USA). GAPDH (1:3,000, Santa Cruz Biotechnology, Santa Cruz, CA, USA.) was employed as the loading control.

### Statistical analysis

Statistical analyses were performed using SPSS version 13.0 (SPSS, Chicago, IL, USA), and all data are presented as mean ± S.D. Statistical differences among the groups were assessed by one-way ANOVA and post hoc multiple comparisons were performed using Student-Newman-Keuls tests. The significance level was set at *p* < 0.05.

## Results

### Isolation and characterization of MSCs

**Figure 1:**
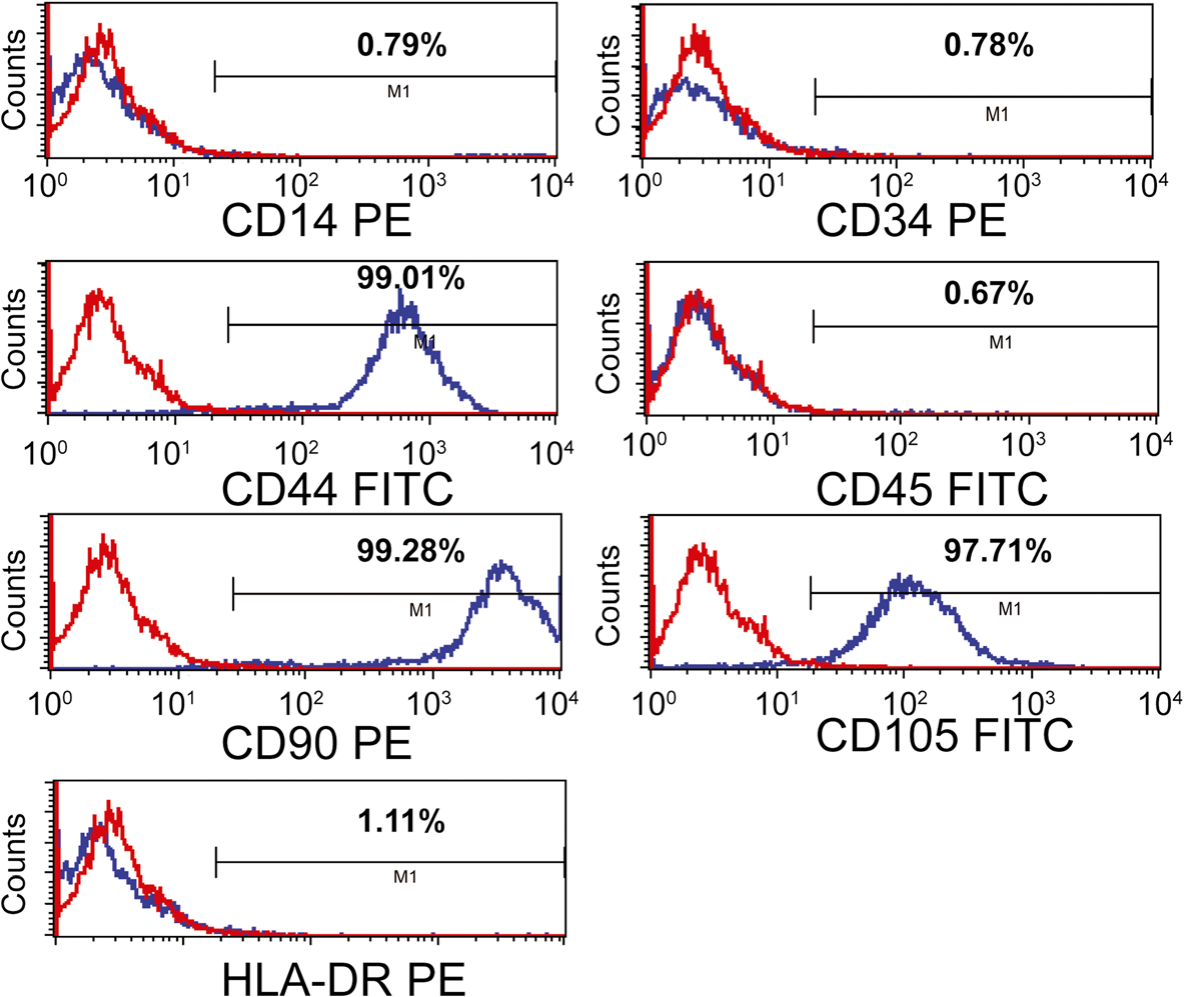
**Surface marker expression in MSCs.** MSCs were confirmed by flow cytometry analysis after three passages as positive for CD44 (99.01%), CD90 (99.28%) and CD105 (97.71%), with low positivity for CD14 (0.79%), CD34 (0.78%), CD45 (0.67%) and HLA-DR (1.11%).

MSCs were isolated from SD rats’ bone marrow and maintained in culture for several passages. Before intravenous transplantation, third- and eighth-passage cells were characterized, and flow cytometry analysis confirmed that the cells at transplantation were positive for CD44 (99.01%), CD90 (99.28%) and CD105 (97.71%), and had low expression of CD14 (0.79%), CD34 (0.78%), CD45 (0.67%) and HLA-DR (1.11%) (Figure 1).

### Treatment with MSCs improved neurological recovery after TBI

**Figure 2:**
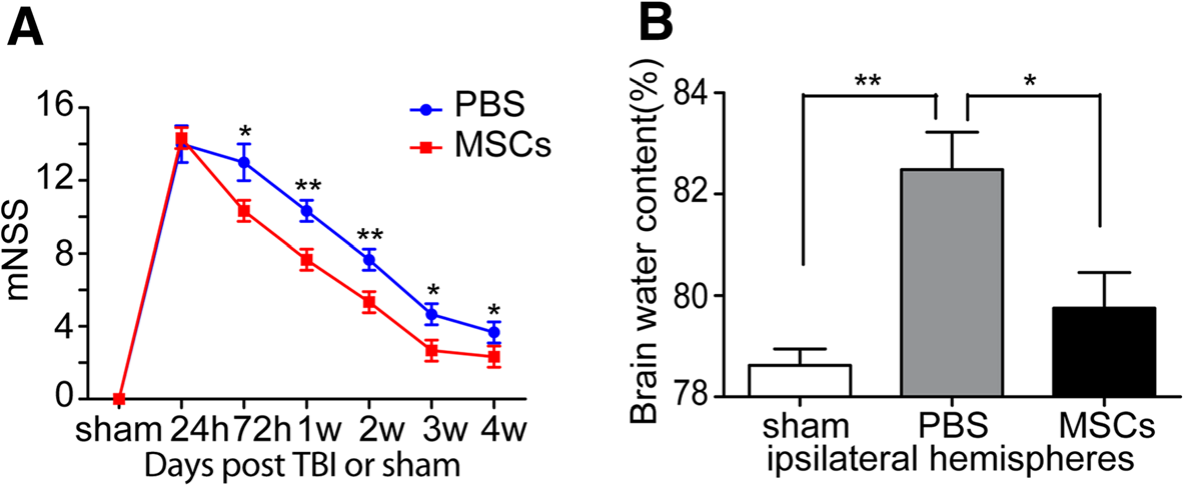
**Modified neurologic severity score (mNSS) and brain water content. (A)** Neurological function was analyzed by mNSS on days 1, 3, 7, 14, 21 and 28 after TBI. Treatment with MSCs significantly lowered mNSS from days 3–28 compared with the PBS group. There was no significant difference in scores between the MSC- and PBS-treated groups only at 24 h post-TBI (*n* = 6 per group). **(B)** Brain water content of ipsilateral hemispheres was measured at 72 h after injury. The PBS group had a significantly higher brain water content than the sham-injured control group. MSC treatment significantly reduced brain water content compared with the PBS group (*n* = 6 per group). Data are presented as the mean ± SD. *p < 0.05, **p < 0.01.

In order to assess the effects of systemic administration of MSCs after TBI, mNSS was performed on days 1, 3, 7, 14, 21 and 28 after TBI. There was a significant improvement in neurological function in the MSC-treated group compared with the PBS group from days 3–28 post-TBI (*p* < 0.05). There was no significant difference between the scores in the two groups only at 24 h post-TBI (Figure 2A).

### MSC treatment reduced brain water content after TBI

To investigate whether treatment with MSCs could reduce brain edema, we measured brain water contents in the two experimental groups. Increased water content directly causes brain edema, which is one of the most important surrogate markers of brain damage, and it peaks at 72 h after brain injury [44]. The PBS group had a significantly higher brain water content than the sham-injured control group. However, treatment with MSCs significantly reduced the brain water contents compared with the PBS group (*p* < 0.05). The brain water contents in the sham, PBS and MSC groups were 78.62 ± 0.32, 82.48 ± 0.74% and 79.87 ± 0.70%, respectively (Figure 2B).

### MSC treatment reduced brain inflammatory cell infiltration, microglia and apoptotic cell numbers

**Figure 3:**
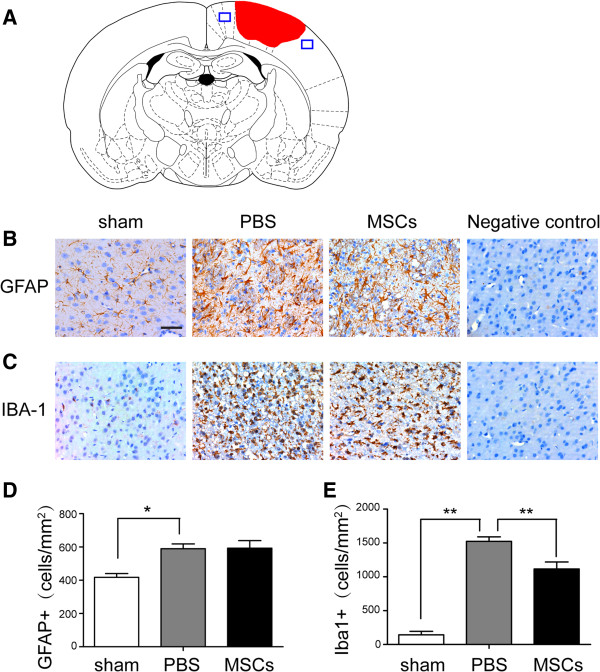
**Effect of MSC treatment on GFAP**^**+ **^**astrocytes and Iba-1**^**+ **^**microglia/macrophages. (A)** Diagram of a coronal rat brain section showing the relationship of the lesion cavity (*red*) to the regions photographed (*blue squares*). The density of astrocytes was not significantly different after MSC treatment **(B**, **D)** (*n* = 6 per group). The number of microglia/macrophages **(C**, **E)** (sham: 142.7 ± 45.4 cells/mm^2^; PBS: 1,524.7 ± 60.1 cells/mm^2^; MSCs: 1,124.3 ± 104.5 cells/mm^2^) was significantly decreased after MSC administration at 72 h post-TBI compared with the PBS-treatment group (*n* = 6 per group). Data are presented as mean ± SD. *Bar* = 50 μm. *p < 0.05.

**Figure 4:**
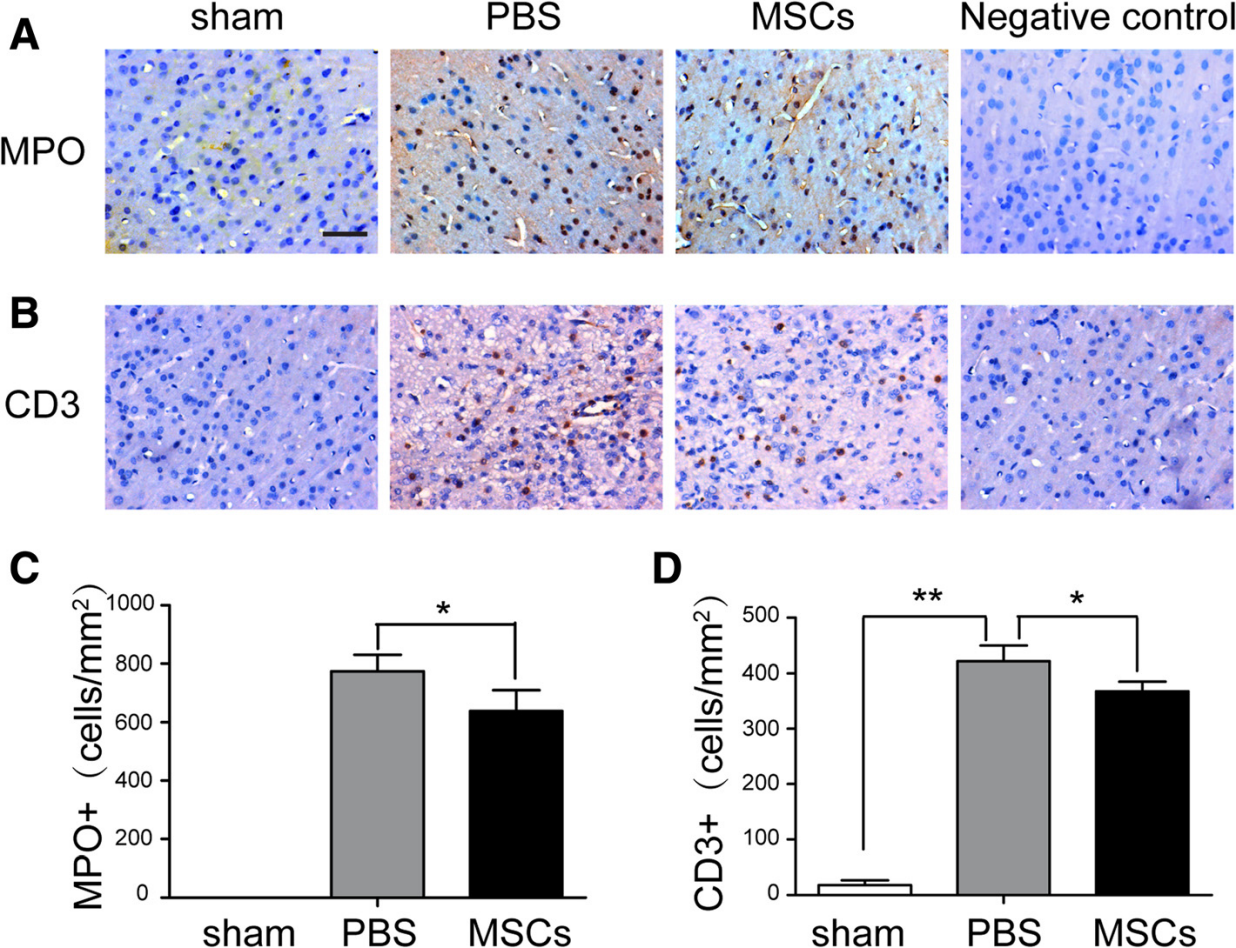
**Influence of MSC administration on MPO**^**+ **^**neutrophils and CD3**^**+ **^**lymphocytes.** MSCs reduced the numbers of infiltrating MPO^+^ neutrophils **(A**, **C)** (PBS: 775.0 ± 55.34 cells/mm^2^; MSCs: 638.67 ± 72.15 cells/mm^2^), CD3^+^ lymphocytes **(B**, **D)** (PBS: 421.67 ± 28.15 cells/mm^2^; MSCs: 367.67 ± 17.5 cells/mm^2^). Data are presented as the mean ± SD. *Bar* = 50 μm; *n* = 6 per group, *p < 0.05.

**Figure 5:**
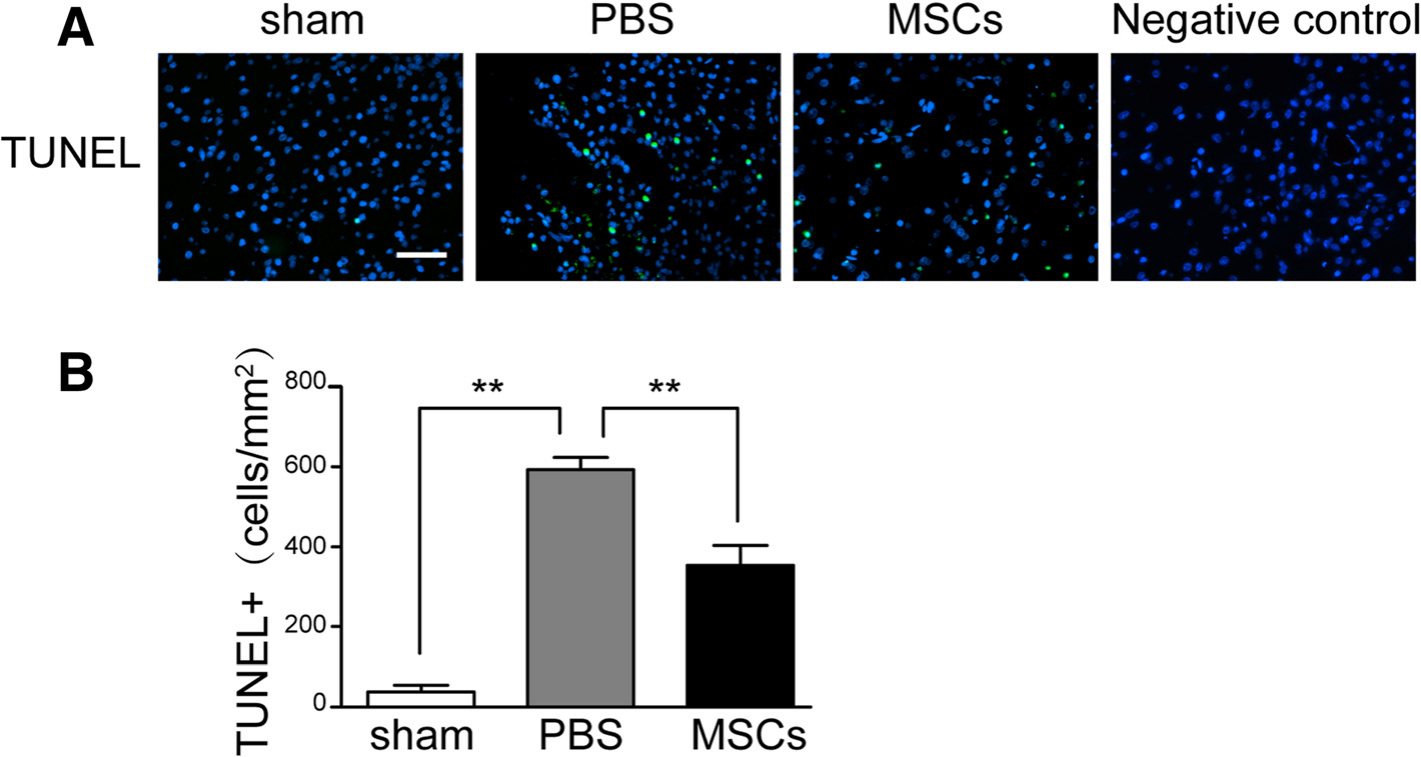
**Effect of MSC transplantation on apoptosis.** Apoptotic cells (sham: 18.7 ± 8.1 cells/mm^2^; PBS: 295 ± 15 cells/mm^2^; MSCs: 179.3 ± 25.8 cells/mm^2^) in the injured cortex at 72 h after TBI were reduced in the MSC treatment group compared with the PBS group **(A**, **B)** (*n* = 6 per group). Number of apoptotic cells is presented as the mean ± SD. *Bar* = 50 μm. **p < 0.01.

To test the effects of MSC treatment on the number of peripheral infiltrating and resident immune cells in the injured brain, we identified GFAP^+^ astrocytes, Iba-1^+^ microglia cells/macrophages, MPO^+^ neutrophils and CD3^+^ lymphocytes by immunohistochemistry. The densities of astrocytes were not significantly different after MSC treatment (Figure 3B,D). The number of microglia/macrophages (sham: 142. 7 ± 45. 4 cells/mm^2^; PBS: 1,524. 7 ± 60.1 cells/mm^2^; MSCs: 1,124.3 ± 104.5 cells/mm^2^) (Figure 3C,E) was significantly reduced after MSC administration at 72 h post-TBI compared with the PBS treatment group (*p* < 0.05). Meanwhile, MSCs decreased the densities of infiltrated MPO^+^ neutrophils (PBS: 775.0 ± 55.34 cells/mm^2^; MSCs: 638.67 ± 72.15 cells/mm^2^) (Figure 4A,C) and CD3^+^ lymphocytes (PBS: 421.67 ± 28.15 cells/mm^2^; MSCs: 367.67 ± 17.5 cells/mm^2^) (Figure 4B,D) and apoptotic cells (sham: 18. 7 ± 8.1 cells/mm^2^; PBS: 295 ± 15 cells/mm^2^; MSCs: 179.3 ± 25.8 cells/mm^2^) (Figure 5) in the injured cortex at 72 h post-TBI (*p* < 0.05).

### MSCs influenced cytokine levels in injured cortex

**Figure 6:**
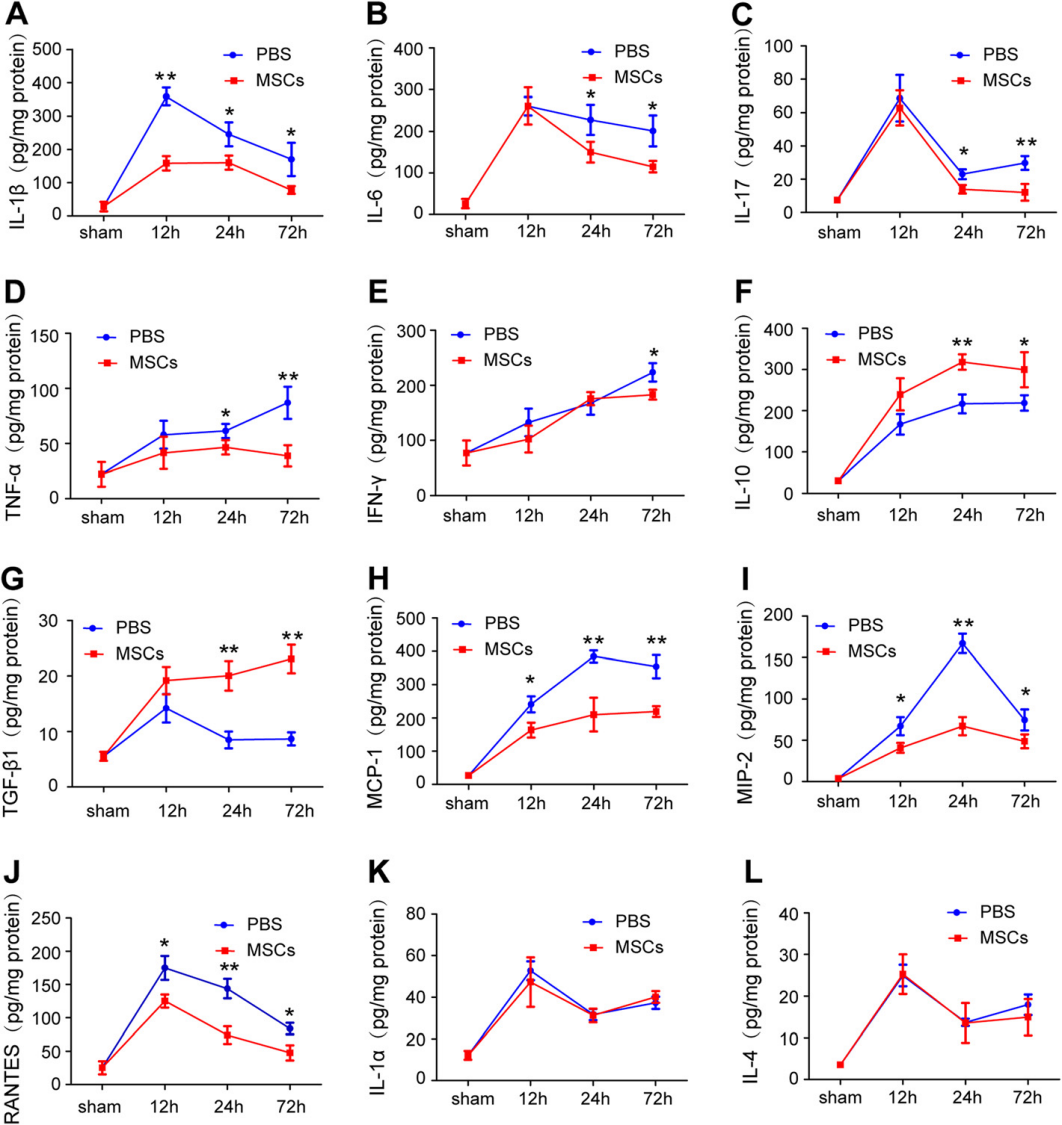
**Influence of MSC treatment on cytokine concentrations.** Levels of the proinflammatory cytokines IL-1β (at 12, 24 and 72 h), IL-6 (at 24 and 72 h), IL-17 (at 24 and 72 h), TNF-α (at 24 and 72 h) and IFN-γ (at 72 h) were significantly decreased in the MSC-treatment group compared with the PBS group **(A**–**E)**. Levels of the anti-inflammatory cytokines IL-10 and TGF-β1 (at 24 and 72 h after TBI) **(F**, **G)** were increased in the MSC-treatment group compared with the PBS group. The chemokines MCP-1, MIP-2 and RANTES were reduced at 12, 24 and 72 h after TBI in the MSC group compared with the PBS group **(H**–**J)**. There were no significant differences in levels of the cytokines IL-1α and IL-4 **(K**, **L)** between the two groups. *n* = 6 in each time point of per group. Data are presented as the mean ± SD. *p < 0.05, **p < 0.01.

To investigate the anti-inflammatory functions of MSCs, we assessed an array of inflammatory cytokines in injured cortex homogenates at 12, 24 and 72 h after TBI (Figure 6). Levels of the proinflammatory cytokines IL-1β at 12 h (*p* < 0.01), 24 h (*p* < 0.05) and 72 h (*p* < 0.05), IL-6 at 24 h (*p* < 0.05) and 72 h (*p* < 0.05), IL-17 at 24 h (*p* < 0.05) and 72 h (*p* < 0.01), TNF-α at 24 h (*p* < 0.05) and 72 h (*p* < 0.01), and IFN-γ at 72 h (*p* < 0.05) were all significantly decreased in the MSC-treatment group compared with the PBS group (Figure 6A-E). In contrast, production of the anti-inflammatory cytokines IL-10 at 24 h (*p* < 0.01) and 72 h (*p* < 0.05) and TGF-β1 at 24 h (*p* < 0.01) and 72 h (*p* < 0.01) (Figure 6F,G) after TBI were increased in the MSC-treatment group compared with the PBS group. The chemokines MCP-1, MIP-2 and RANTES were reduced at 12, 24 and 72 h after TBI in the MSC group compared with the PBS group (Figure 6H-J). There were no significant differences in levels of the cytokines IL-1α and IL-4 (Figure 6K,L) between the two groups.

### MSC treatment upregulated TSG-6 expression

**Figure 7:**
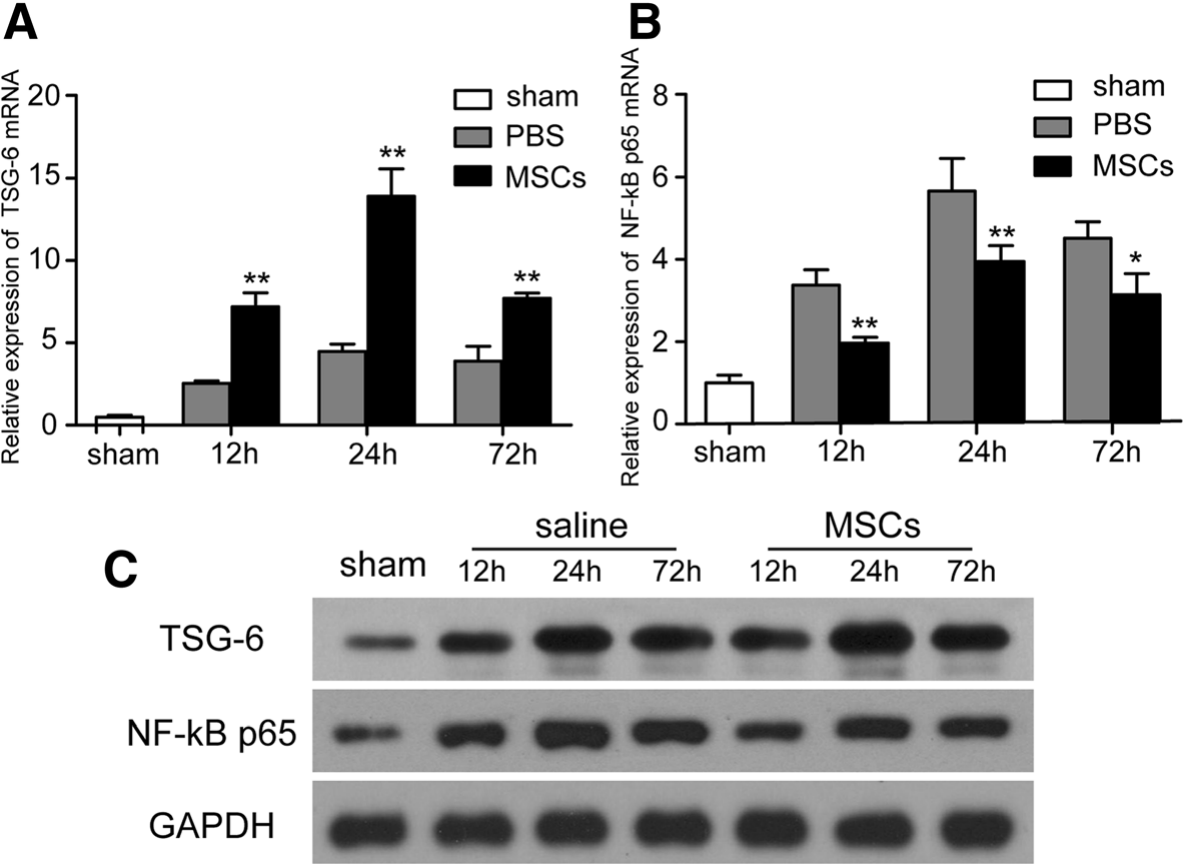
**MSC treatment upregulates TSG-6 and downregulates NF-κB expression.** Upregulation of TSG-6 **(A)** was observed from 12 to 72 h in the injured cortex after TBI in the MSC-treatment group. mRNA levels of NF-κB **(B)** decreased from 12 to 48 h after MSC transplantation. Similar results were observed at the protein level **(C)** for TSG-6 and NF-κB p65. *n* = 6 in each time point of per group. Data are presented as the mean ± SD. *p < 0.05, **p < 0.01 versus PBS group.

To elucidate the potential mechanisms responsible for the effects of MSCs on anti-inflammatory and immunomodulatory properties, we analyzed the expression of the inhibitory factors TSG-6 and transcription factor NF-κB at the mRNA and protein levels. TSG-6 was upregulated from 12–72 h in the injured cortex after TBI in the MSC-treatment group (*p* < 0.01) (Figure 7A). mRNA levels of NF-κB (Figure 7B) were decreased from 12–48 h after MSC transplantation (*p* < 0.01). Similar results were obtained by Western blotting for TSG-6 and NF-κB p65 (Figure 7C).

## Discussion

In this study, we investigated the anti-inflammatory and immunomodulatory properties of MSCs by systemic transplantation into TBI model rats. The main observations were that MSC treatment reduced the presence of microglia/macrophages in the damaged brain parenchyma and decreased the density of peripheral infiltrating leukocytes at the injured site, as well as reducing proinflammatory cytokines and increasing anti-inflammatory cytokines, possibly through enhanced expression of TSG-6. TSG-6 may, in turn, act by suppressing activation of the NF-κB signaling pathway and decreasing the production of proinflammatory cytokines to initiate a proinflammatory cytokine cascade.

Proinflammatory cytokines such as TNF-α, IL-1 and IL-6 are produced mainly by microglia, with some also produced by astrocytes, neurons and endothelial cells, which in turn activate glial cells, inducing further cytokine production and astrogliosis [4,45,46]. Reduced activation of microglia can thus reduce inflammation and improve histological and functional outcomes after TBI [8]. Anti-inflammatory cytokines such as IL-4, IL-10 and TGF-β1 have the ability to counteract and downregulate inflammatory and cytotoxic reactions [4,47]. For instance, IL-10 is produced by microglia and astrocytes and by lymphocytes in the periphery, and can suppress microglia and astroglia activation, as well as decreasing production of proinflammatory cytokines [48,49]. MSCs have been shown to decrease proinflammatory cytokine gene expression in experimental acute lung injury [31,32], myocardial infarction [33] and acute renal failure [34] and to upregulate IL-10 expression in rat models of myocardial infarction and cerebral infarction [50,51]. Although astrocytes are not directly immune-related cells, activated astrocytes are a major source of inflammatory-related molecules such as pro- and anti-inflammatory cytokines and chemokines. In addition, astrocyte activation and proliferation after TBI seem to impair axonal regrowth, but these cells also release neurotrophic factors promoting tissue repair and neurogenesis [52]. However, the immunomodulatory effects of MSCs on astrocytes are still limited. One study has shown that MSCs can inhibit the production of cytokines in LPS-activated astrocyte cultures, reducing not only the proinflammatory cytokines, but also the expression of the anti-inflammatory IL-10 [53].

In the CNS, chemokines secreted by glia and neurons are considered to be essential mediators in the recruitment of leukocytes into damaged parenchyma in post-traumatic neuroinflammation [43]. MIP-2, also known as CXCL2, contributes to neutrophil infiltration and subsequent secondary neurodegeneration following TBI [54], and it has been shown to increase rapidly following TBI in experimental TBI models [55,56]. MCP-1, also known as CCL2, is the most potent chemoattractant for monocytes, macrophages and microglia, which play significant roles in mediating post-traumatic secondary brain damage [43]. CCL2 expression is elevated rapidly after diffuse axonal injury, and its overexpression exacerbated ischemic brain injury in mice [57,58]. Expression of the chemokine RANTES (regulated upon activation, normal T cell expressed and secreted) increased after brain injury in rats and has the ability to activate T cells [59]. The role of T lymphocytes in TBI is largely unknown, although in ischemic stroke, these cells infiltrate into the brain and release proinflammatory cytokines and cytotoxic substances, which contribute to early inflammation and brain injury [10]. One study has shown that reduction of T-lymphocyte recruitment significantly enhances tissue preservation and functional outcome after spinal cord injury [60]. Therefore, reduction of neutrophil and T lymphocyte infiltrations at an early stage is a key feature in improving TBI outcome [61]. In contrast, T lymphocytes can also have beneficial effects on the repair and regeneration of the brain at later stages following injury [10]. Our results indicated that MSCs reduce production of the chemokines MIP-2, MCP-1 and RANTES, suggesting that they could act through reducing chemokine production, thus decreasing the recruitment of peripheral leukocytes.

The anti-inflammatory and immunosuppressive effects of MSCs were related to several inhibitory factors such as inducible nitric oxide synthase, indoleamine 2,3-dioxygenase, prostaglandin E2 and TSG-6, which are produced by MSCs or released following cross-talk with target cells, and which have been reported to be involved in MSC-mediated immune regulation [22]. The current study did not investigate inhibitory factors produced by MSCs, such as inducible nitric oxide synthase, indoleamine 2,3-dioxygenase or prostaglandin E2, because their short half-lives mean that their immunosuppressive effects can only be observed *in vitro* or because they have adverse effects when administered systemically [62]. TSG-6 is an anti-inflammatory protein with multiple anti-inflammatory effects that is induced by the inflammatory cytokines TNF-α and IL-1 [63]. Transplanted MSCs played a crucial role in the suppression of inflammation in models of myocardial infarction and corneal injury, and these anti-inflammatory effects may be attributable to the secretion of TSG-6 by MSCs [33,64]. NF-κB is an important transcription factor that regulates many genes with key roles in immune and inflammatory responses. It is activated in the brain after TBI and contributes to neuronal death [65,66]. Inhibition of NF-κB activation may thus reduce adverse inflammatory response events and reduce the loss of neuronal cells after TBI. TSG-6 can reduce the production of proinflammatory cytokines through suppressed activation of the NF-κB signaling pathway, thus initiating the cascade of proinflammatory cytokines [67]. Our results suggest that the beneficial effects of MSCs may be partially explained by the effect of TSG-6 on the NF-κB pathway.

The results of this study raise several issues regarding the clinical use of MSCs. First, the schedule of MSC administration is important. MSCs are not spontaneously immunosuppressive and only exhibit this property under special conditions [22]; stimulation with certain inflammatory cytokines such as IFN-γ is essential for MSC-mediated immunosuppression [68]. If the levels of inflammatory cytokines are too low, the immunosuppressive effect of MSCs will not be triggered. Inflammatory cytokines are rapidly upregulated following brain injury, and some peak at as little as 2 h after TBI [7]. In addition, neutrophil and T lymphocyte infiltrations peak at 24 h after TBI [61], and microglia activation is induced immediately after injury [69,70]. Later MSC treatment may therefore not be effective in terms of anti-inflammatory or immunosuppressive activity, while MSC administration at the onset of the inflammatory response after TBI is more likely to be effective. In this study, we therefore transplanted MSCs at 2 h after TBI. Second, the route taken by the cells is also important. Previous studies showed that in the case of intravenously injected MSCs, few cells reached the brain parenchyma after the pulmonary first-pass effect [16]. The ideal treatment combined intravenous injection with direct injection to provide both systemic and local therapeutic effects [21]. Finally, multiple administrations of MSCs may be needed to sustain and prolong their inhibitory effects, as demonstrated in a mouse graft-versus-host disease model [71].

MSCs are known to modulate both systemic and local inflammation systems in neuroinflammation [72]. We did not investigate the effect of MSCs on systemic inflammation, but the possibility that the neuroprotective effects of MSCs are caused by modulation of the systemic inflammatory system cannot be ruled out. Recent evidence suggests a link between brain injury and the autonomic release of proinflammatory cytokines by resident macrophages in the spleen [73]. Inhibiting this release by splenectomy was shown to improve outcomes in animal models of TBI [44], implying that transplanted MSCs acting directly on resident macrophages in the spleen could contribute to the neuroprotective effect. Overall, these results suggest that novel MSC-mediated immunosuppression mechanisms may be developed for the therapy of TBI.

## Conclusions

These results suggest that MSCs have the ability to modulate inflammation-associated cytokine release and immune cells in TBI-induced cerebral inflammatory responses. This study serves as the basis for future studies and offers new insights into the mechanisms responsible for the beneficial immunomodulatory effect of MSC transplantation in terms of functional neurological recovery after TBI. However, further studies are needed to resolve outstanding issues regarding the clinical use of MSCs in TBI.

## Abbreviations

MSC: Mesenchymal stem cell; TBI: Traumatic brain injury; CNS: Central nervous system; mNSS: Modified neurologic severity score; TSG-6: Stimulated gene/protein 6; NF-κB: Nuclear factor-κB.

## Competing interests

The authors declare that they have no competing interests.

## Authors’ contributions

Conceived and designed the experiments: RZ, XDJ. Performed the experiments: RZ, YL, KY, XRC, LC. Analyzed the data: FFC, PL. Wrote the paper: RZ, XDJ. Paper revision: XDJ. All authors read and approved the final manuscript.
